# The dimensionalities of lesion-deficit mapping

**DOI:** 10.1016/j.neuropsychologia.2017.09.007

**Published:** 2018-07-01

**Authors:** Tianbo Xu, Ashwani Jha, Parashkev Nachev

**Affiliations:** a Institute of Neurology, UCL, UK; b National Hospital for Neurology and Neurosurgery, Queen Square, UK

**Keywords:** Lesion-deficit brain mapping, Structural neuroimaging, Multivariate analysis, Machine learning, Stroke

## Abstract

Lesion-deficit mapping remains the most powerful method for localising function in the human brain. As the highest court of appeal where competing theories of cerebral function conflict, it ought to be held to the most stringent inferential standards. Though at first sight elegantly transferable, the mass-univariate statistical framework popularized by functional imaging is demonstrably ill-suited to the task, both theoretically and empirically. The critical difficulty lies with the handling of the data's intrinsically high dimensionality. Conceptual opacity and computational complexity lead lesion-deficit mappers to neglect two distinct sets of anatomical interactions: those between areas unified by function, and those between areas unified by the natural pattern of pathological damage. Though both are soluble through high-dimensional multivariate analysis, the consequences of ignoring them are radically different. The former will bleach and coarsen a picture of the functional anatomy that is nonetheless broadly faithful to reality; the latter may alter it beyond all recognition. That the field continues to cling to mass-univariate methods suggests the latter problem is misidentified with the former, and that their distinction is in need of elaboration. We further argue that the vicious effects of lesion-driven interactions are not limited to anatomical localisation but will inevitably degrade purely predictive models of function such as those conceived for clinical prognostic use. Finally, we suggest there is a great deal to be learnt about lesion-mapping by simulation-based modelling of lesion data, for the fundamental problems lie upstream of the experimental data themselves.

## Highlights

• Neglecting two dimensionalities—lesion and functional anatomy—distorts lesion maps.
• Lesion dimensionality is critical, mandating high-dimensional multivariate methods.
• Simulations based on real lesion data applied to synthetic ground truths are needed.
• Anatomical fidelity matters for clinical outcome prediction as for spatial inference.

## 1 Introduction

In common with all scientific inference, the fidelity of lesion-deficit mapping depends on the quality of the source data and the validity of the models applied to it. Though equally important, the two aspects are sharply distinct: a deficit in neither is remediable by an excess of the other. Whereas a good model may be improved by better data, a defective model is often irredeemably so. The validity of a model is judged by hard, logico-mathematical criteria, the quality of data by softer, empirical opinion. Inferential failure resulting from poor data tends to be graceful, proportionate with the degree of data corruption; by contrast, model errors may have catastrophic consequences even when seemingly minor. Worse, failure from a defective model is often *silent*, cloaked in superficially attractive significance values that conceal fatal *biases* in the inference repetition can only entrench. Where no other inferential technique is stronger, such systematic errors may easily persist indefinitely.

Why do we need reminding of these statistical platitudes? The hazards of modelling are greatest where the complexity of the system under study is highest, as is archetypally true of the brain. For our purposes it suffices to define complexity as the minimum number of dimensions required to predict one state of a system from another: its *intrinsic dimensionality*. If our models cannot be commensurately complex—for reasons of intellectual opacity or computational tractability—it is tempting aggressively to simplify them, for then the unmodelled signal superficially resembles noise. But if the residual variance retains appreciable structure, the inference will be distorted in ways the simplicity of the model merely conceals from view. The inevitable inferential distortion aside, the more non-stochastic variability the model does not explain, the weaker its explanatory power, and—of course—its practical, clinical utility.

So how do we determine the correct dimensionality? A perfect answer is impossible, for it assumes precisely the knowledge our models are deployed to acquire. But we can examine the grounds for an informed supposition, and we can also explicitly *test* the consequences of adopting one solution over another. Here we give the empirical and conceptual grounds for our view on the necessary dimensionality, and go on to outline the explicit tests one ought to conduct to confirm or infirm it. Although this is certainly not the only important methodological concern in lesion-deficit mapping, we dwell on it at length here because it has received so little of the attention it requires.

## 2 The dimensionality of anatomical inference in the brain

Let us first clarify how the dimensionality of an inferential model is determined. Formal lesion-deficit mapping began with taking the overlap of a set of lesions and contrasting the peak with that derived from another, control set of patients (e.g. (Robertson et al., 1988)). Such a comparison is produced by a simple voxel-wise operation that ignores any anatomical relationship but that between homologous voxels across the two groups. This is mass-univariate inference, even if it was not called so at the time, for the contribution of each voxel is independently quantified by its own, univariate test, whether implicit or explicit. By replacing simple subtraction with a formal statistical test, voxel-wise lesion-symptom mapping (VLSM) and kindred techniques add a measure of confidence to the inference at each voxel, leaving the independence assumption untouched, and the inference univariate (Bates et al., 2003; Chen and Herskovits, 2010; Damasio et al., 1996; Karnath et al., 2004; Rorden et al., 2007). Additional variables may be added to the voxel-wise statistical test—various behavioural covariates, for example—making it multivariate, but *not* from the critical perspective of the anatomy, for that is still modelled as a set of independent locations, evaluated over multiple statistical tests run at each voxel in isolation from every other. So this is still mass-univariate *anatomical* inference, even if its *behavioural* dimensionality may be expanded.

Now two manoeuvres here commonly escalate the anatomical dimensionality. The most common is the addition of lesion volume as a covariate, a crude index of damage at other voxels (Karnath et al., 2004). This attempts to capture the effect on behaviour of the global change in available brain substrate, independently of anatomical location, reasoning that parcelling out such anatomically non-specific effects will increase sensitivity for the anatomically specific effects of interest. Less common is the use of Gaussian smoothing, which changes the value of a voxel on the assumption is relation to its neighbours is adequately described by a random Gaussian field (Kimberg et al., 2007). Since neither is capable of conveying any substantial anatomical detail, most would still regard such models as mass-univariate. Moreover, we still have one model per voxel, and therefore as many models as there are estimated voxels in the brain.

An analysis becomes anatomically multivariate where the statistical model incorporates many anatomical variables, indexing the presence or absence of damage to different parts of the brain *together* (Chen et al., 2008; Chen and Herskovits, 2015; Keinan et al., 2004; Mah et al., 2014; Rondina et al., 2016; Smith et al., 2013; Toba et al., 2017; Yourganov et al., 2016; Zhang et al., 2014). The dimensionality of such models depends on the number of such variables and their properties. Where the variables are correlated, the intrinsic dimensionality will be less than their number, but this is usually something to be established by the analysis itself, implicitly in the inferential model, or explicitly in a preceding dimensionality reduction step. Either way, each inferential model now covers all or a substantial part of the brain, leaving us with one or few models per brain where a multiplicity of voxels describe a large number of dimensions per model.

Naturally, the dimensions of behaviour and anatomy are bound to interact, and a model may be critically deficient in either or both. Our focus here is on the anatomical not because the others should be neglected but because the anatomical near-universally have.

## 3 Two determinants of dimensionality: brain and lesions

It is natural to think of anatomical factors as pertaining only to the functional architecture of the brain. But in lesion-deficit mapping this is only one side of the coin: there is a second anatomical dimensionality to consider, that arising from the lesion architecture. We need to examine each in turn.

### 3.1 Brain dimensionality

That Lego^®^ is not helpfully metaphorical of the brain's functional architecture is increasingly recognized in the emphasis on highly distributed operations subserved by complex, dynamic functional networks (Sporns et al., 2005). Both disruptive and correlative data unequivocally point to an underlying neural organisation in which complex *interactions* between areas determine the observed behaviour (Young et al., 2000). Such interactions may be non-monotonic, reflective of neural relations that could just as easily be competitive as collaborative. They are—moreover—bound to be *adaptive*, varying across both time and individuals. An entire field of clinical neuroscience—functional neurosurgery—richly illustrates these truths in each and every patient, where *disruption* of one area of the brain—optimised both within and across patients—is used to improve the function of the brain as a whole (Jha and Brown, 2011, Johnson et al., 2008).

A satisfactory model of a lesioned brain must therefore not only model the individual functions of the affected areas but their—potentially highly complex—interactions. The syndrome of visuospatial neglect offers a striking example of this: neglect caused by damage to inferior parietal areas may not only *not* be exacerbated by damage to the contralateral frontal eye field but wholly *reversed* by it (Vuilleumier et al., 1996). It is obvious that in evaluating the lesion-deficit relationship in a patient we must here model the presence and absence of damage at *both* loci, *together*, and if this is true of this particular pair it may be true of any combination of areas, across the entire brain (Price and Friston, 2002; Zavaglia and Hilgetag, 2016).

The optimal lesion-deficit model, then, is one in which the integrity of each functionally homogeneous location in the brain is a separate variable. Since no wholly convincing definition of functional homogeneity is currently available (pace (Glasser et al., 2016)), our limit becomes practical: such anatomical parcellation of the brain as our tools can provide, minimally the voxel size of the imaging acquisition. Anything short of this will miss interactions at a finer level of anatomical organisation. Even with voxel sizes of remarkable coarseness—8 mm isotropic—this leaves us with several thousand variables per brain: a high-dimensional model, certainly in proportion to the number of patients included in the typical lesion-deficit study.

### 3.2 Lesion dimensionality

The variable expansion we are discussing here is driven by the dimensionality of the functional architecture. But in lesion-deficit mapping there is a second, independent dimensionality to consider: that of the lesion architecture (Mah et al., 2014, Mah et al., 2015). Where lesions overlap—and are generally larger than the minimal size of functionally homogeneous areas—the lesion-deficit relation will be influenced by both the functional and the lesion architecture. This is overwhelmingly true of the lesions described in the current literature, a reflection of the natural characteristics of the underlying pathology, especially the commonest: vascular injury.

Let us consider carefully why the lesion architecture matters here. In functional imaging, the physiological cause of the change in the BOLD signal operates at sub-voxel granularity, for it is driven by the microvasculature (Logothetis et al., 2001). Such anatomical structure as emerges at the voxel level is then plausibly related to the underlying neural anatomy, even if there may well be non-linearities in the relation between BOLD and neural activity across the brain (Birn et al., 2001, Heeger and Ress, 2002). If two voxels are co-activated it will *not* be because an idiosyncrasy of the microvasculature makes it so, for the vascular causal mechanisms do not operate at that anatomical scale. Consider, by contrast, lesion-deficit mapping, where the effective equivalent of BOLD activation is a lesion, almost invariably extending across multiple voxels as an outcome *not* of the underlying functional anatomy but of the causal pathological process. The anatomy of the lesion is highly unlikely to be random, for the underlying pathological process rarely is: if two voxels are damaged together, it is because a pathological process—exhibiting its own structured characteristics—has caused it.

These characteristics will vary from one pathological process to another. In the case of the commonest macroscopic focal brain pathology—ischaemic injury—they will inevitably reflect the complexity of the macrovascular architecture of the brain. Without a satisfactory means of registering vascular trees and modelling their interaction with the various forms of pathological stenosis and occlusion, we cannot easily arrive at a sharp estimate, but it is wildly implausible the characteristics of the resultant lesions will be either simple or perfectly random. Other common pathologies—neoplasms and traumatic injuries—will similarly produce lesions no simpler than the complex processes that cause them. Unlike functional imagining, then, in lesion-deficit mapping we must also take into account the patterns of covariance across voxels resulting from the underlying pathology: what we have termed parasitic voxel-voxel associations. This problem is also likely to be intrinsically high-dimensional, for the pathological processes are irreducibly complex.

There are then two, independent, *a priori* reasons for adopting a high-dimensional approach: the complexity of the functional architecture of the brain, and the complexity of the anatomical structure of lesions. Since the inferential consequences of neglecting each kind of complexity are radically different, it is crucial we understand the differences between them. Most researchers are justifiably relaxed about the former, and alarmingly unaware of the dangers of the latter.

## 4 The consequences of neglecting dimensionality

Let us imagine a new disease—something resembling neurocysticercosis (Garcia and Del Brutto, 2005)—that inactivates discrete volumes of tissue exactly the size of a single voxel randomly drawn from a uniform spatial distribution. Here the lesion architecture is definitionally random, and so cannot be expected to introduce a significant bias in any inference. The dimensionality we need to be concerned about therefore reduces to that of the functional architecture itself. What happens if we ignore it, following conventional mass-univariate functional imaging and the lesion-deficit mapping inspired by it?

Functions dependent on single, contiguous, anatomically-invariant areas, will be mapped with spatial precision that will monotonically increase with sample size, in exact analogy with functional imaging. The greater the variability of the critical anatomical location the less precise will be the resultant map—and consequently its individual predictive performance—but the limit is here merely the inter-subject variance on which the very conception of a population map is premised.

Functions dependent on multiple, discrete areas, will be mapped with spatial precision that will also monotonically increase with sample size, but will be modulated by the nature of their functional interactions. Where this is adequately described by an OR operator—e.g. a syndrome results from damage to area X or area Y—identifying each area will naturally be harder, for a model of one critical area will incorrectly "see" instances of damage to another as false negatives. Where the operator is an AND, the problem will be the converse: the model of each area will see instances of damage local to it as false positives. But in both cases, although a map may take more samples to define, its precision will also be bounded merely by the inter-subject variability in the anatomical locations of the critical areas, exactly as with functional imaging. And having identified multiple, spatially discrete areas, a researcher would naturally be subsequently drawn into exploring their interactions, implicitly escalating the dimensionality of the model as functional imagers do when they model psychophysiological interactions. Of course, if having identified just one area from an inadequate sample the researcher stops collecting data and goes to print, a spatial bias may nonetheless emerge, but as a consequence less of the inferential framework than of the infelicities of wider scientific practice.

In short, neglecting interactions between functional areas makes lesion-deficit mapping harder, but not necessarily less accurate. Dimensional poverty is here acceptable, and though greater wealth is desirable it would be nouveau riche to *insist* on it.

Now consider the converse of the preceding imagined example: an organisation of the brain where a given function depends on a single voxel—disabled when it is hit, normal when it is intact—but where the lesions used to map it exhibit the complex architecture seen in clinical reality. Here the functional architecture is definitionally simple, and so cannot be expected to introduce a significant bias in any inference. The dimensionality of concern becomes only that of the lesion architecture. What happens if we ignore *this* dimensionality, as conventional mass-univariate inference does?

**Fig. 1:**
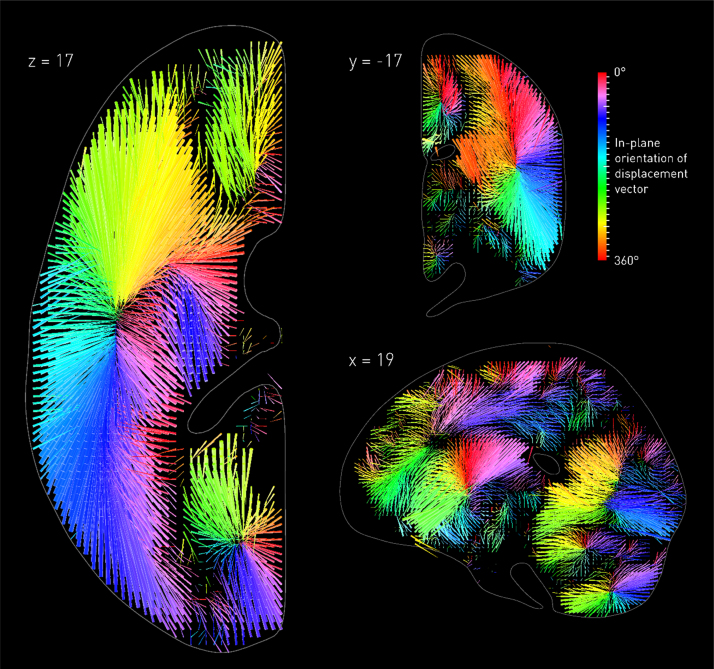
**The biasing effects of the vascular tree.** Three-dimensional vector plot of the direction (colour map) and magnitude (length of arrow) of mislocalization at adequately sampled voxels within three representative planes (left axial, top coronal, bottom sagittal), based on a sample of 581 acute stroke lesions, normalized into standard stereotactic space and mirrored onto one hemisphere (see Mah et al., 2014 for details). The value at each voxel was calculated by labelling the stack of 581 lesioned volumes as being ‘affected’ or ‘unaffected’ depending on whether or not that voxel fell within the lesion in each volume, running a standard voxel-wise Fisher's exact test-based mass-univariate analysis on the two groups, and identifying the centre of mass of the resultant significant cluster, identified by the asymptotic p-value thresholded at a Bonferroni corrected p < 0.01. This procedure was performed at all voxels hit more than three times in the data set. Each arrow points from the true location of a voxel in the brain to the location where the mass-univariate model erroneously places it. The colour map corresponds to the orientation of this error vector in the visualised plane. Note that the mislocalization tends to follow the organisation of the vascular tree, with clusters corresponding to the branches of the middle cerebral, anterior cerebral, and posterior circulations. (For interpretation of the references to color in this figure legend, the reader is referred to the web version of this article.)

Whenever a target voxel is hit, *other* voxels will be collaterally hit with it, as shaped by the covariances naturally introduced by the pathological process. Though irrelevant, these voxels will be *parasitically* associated with the function, in a manner dictated by the lesion—not the functional—architecture. The estimated cluster of significantly associated voxels will then contain not just the target voxel but potentially many others, following a pattern that need neither centre on the voxel nor exhibit any functionally-determined relation other than enclosing it. Crucially, this relation *will not be random*, for the pathological process determining it is not random. Within the frequentist framework in commonest use, there are no grounds for favouring one voxel crossing the significance threshold over another, but even within a Bayesian framework voxels with perfect collaterality will be indissociable. The centroid of that cluster may therefore be far from the critical voxel. The resultant mislocalisation will only be entrenched with repetition, for the effect arises not from noise but from an unmodelled source of spatial *bias* in the lesion data. So even a model of the brain of utterly implausible simplicity—invariant single-voxel dependence—will consistently mislocalise when the complexity of the lesion architecture is neglected (Fig. 1) (Mah et al., 2014).

If we complicate our model of functional anatomy to the regional—rather than single-voxel—dependence closer to reality, the resultant mislocalisation can only be worse, for now distinguishing between critical and parasitic voxels becomes much harder. Moreover, a set of irrelevant, collaterally damaged voxels will be *more* strongly associated with the deficit if damage to no part of a larger set of actually critical voxels shows comparable or lesser variability. The most significant cluster here need not include *any* part of the critical region at all, let alone its totality. And if we now complicate our model even further to include interacting networks of remote areas—the only biologically plausible organisation of the brain—it should be obvious the biasing effects can only be massively amplified. Given the known complexity and range of white matter connectivity, such remote interactions could never be modelled simply, and certainly not merely by proximity (Herbet et al., 2015, Mah et al., 2015). And the more distributed the anatomy, the more of the inferred lesion-deficit structure will be driven by the lesion architecture, for its biasing influences are then given even freer reign.

In short, ignoring the complexity of the lesion architecture does not make lesion-deficit mapping harder, it invalidates it completely. Dimensional poverty is here is simply *not* acceptable, for all we can say of low-dimensional models is that they are certain to be misleading. *This* is the principal reason for insisting on a high-dimensional approach in lesion-deficit mapping, not the distributed nature of the functional architecture of the brain (Table 1).

**Table 1:** The dimensionalities of lesion-deficit inference. The inferential consequences of neglecting the dimensionality of lesion-deficit data vary depending on the source. Where the anatomy is complex, mass univariate methods are inefficient but not necessarily biased. By contrast, where the lesion anatomy is complex, mass univariate methods become unquantifiably biased. High-dimensional multivariate inference solves both problems, but is conditional on the availability of large datasets. Note what constitutes sufficient data is difficult to prescribe, for conventional power analyses are not applicable here: the only guide is behavioural predictive performance of the anatomical model, tested on independent, “out-of-sample” data.

	*Dimensionality of functional anatomy*	*Dimensionality of lesion anatomy*
*Mass-univariate inference*	Insensitive but spatially mostly unbiased, given sufficient data	Spatially biased, regardless of data size.
*High-dimensional multivariate inference*	Sensitive and spatially unbiased, given sufficient data	Sensitive and spatially unbiased, given sufficient data

## 5 Evaluating lesion-deficit models with synthetic ground truths

Were switching to high-dimensional modelling simply a matter of adding another toolbox to one's MATLAB path, we need do no more than draw attention to the foregoing. But one cannot easily escalate the dimensionality of a model without increasing the minimal size of the datasets needed satisfactorily to estimate it. High-dimensional methods often require machine learning based on exotic mathematics outside the comfort zone of many statisticians, let alone biologists, and may constitutionally lack easy interpretability anyway (Angermueller et al., 2016). In a field where securing even modest cohorts of patients is a challenge, and where the focus of intellectual attention is generally far from novel computational methods, it is easy to perceive insisting on such a change as destructive, nihilistic even.

So however inevitable conceptually, we need to demonstrate the need for change empirically. What form should such a demonstration take? We must use real lesion data, for we are concerned with its empirical properties. But we cannot easily use real functional anatomy when this is what we use lesion mapping to determine in the first place. We could use the power to predict individual behaviour as a proxy measure of anatomical fidelity, but such a test has two critical defects. First, it is only one-sided: a high-dimensional model may perform as badly as a low-dimensional one simply because the underlying functional anatomy is too variable, not because the model is not vastly superior. Second, even when correctly estimated, a high-dimensional model may sometimes achieve relatively high individual predictive power without adequately identifying the underlying causal anatomical features.

Rather than use real functional anatomy here it is far better to define a synthetic, artificial ground truth. This gives us full control over the space of possible anatomical organisations, allowing us to quantify the mapping error for arbitrarily large families of different lesion-deficit models (Inoue et al., 2014; Mah et al., 2014; Pustina et al., 2017; Sperber and Karnath, 2017; Zhang et al., 2014). That our models will likely be simpler than reality is not a critical defect, for we are interested in quantifying the *minimal* error: the "best case" scenario. If *this* error is large, then the reality can only be worse. Where such an analysis is combined with a lesion dataset far larger than is generally used in lesion-deficit studies, the error cannot plausibly be trivially attributed to relative undersampling, and so its quantification becomes authoritative.

Though powerful—indeed key to further progress in the field—such modelling has rarely been applied to the problem of dimensionality in lesion-deficit mapping. But we can use the five published studies (one available only in pre-print form) to highlight the distinctive features and limitations of the approach (Inoue et al., 2014, Mah et al., 2014, Pustina et al., 2017, Sperber and Karnath, 2017, Zhang et al., 2014).

First, the critical output of interest of a synthetic lesion-deficit model is the estimated distribution of anatomical mislocalisation, not any qualifying statistic, essentially the *size* of the anatomical error. This tells us how wrong, in millimetres, the spatial inference will on average be, given the underlying model of lesion-deficit relations. That the distribution of another model is statistically different does not matter if the extent of mislocalisation remains substantial. For example, the change in the distributional mean error to 11.5 mm reported by Sperber and Karnath—under idealised, synthetic conditions—still leaves enough error to mislocalise across lobes of the brain: this is miles, in neuroanatomical terms. Reviewing a set of such studies meta-analytically, the salient values are the size and variance of the localisation error across them. In the five independent studies carried out so far, mass univariate approaches are shown to produce errors of similar and very substantial order. Where comparisons with high-dimensional approaches are drawn, the errors are materially—not just statistically—lower (Mah et al., 2014, Pustina et al., 2017, Zhang et al., 2014).

Second, any estimated error distribution will always be specific to the hypothesized lesion-deficit relation. Since the real lesion-deficit relation is definitionally unknown, we must hypothesize a *variety* of them. It is natural to structure this process hierarchically, beginning with simple relations, incrementally escalating to the complexity likely to obtain in reality. A model that performs poorly when the relation is simple *can only be worse* when it is more complex. Making the simplest modelled relation too simple to be biologically plausible ensures any error is not trivially explained by an artificially contrived complexity. Modelling simple relations can also help illustrate the likely *source* of lesion-induced bias: in Mah et al. (2014), for example, revealed by the patterns of distortion to be the anatomical structure of the vascular tree. Though the model has no explicit information about the vasculature, when a single-voxel lesion-deficit is assumed the resultant mislocalisation field clearly identifies the major vascular territories, thereby revealing them to be its principal drivers (Fig. 1).

Crucially, a methodological change that improves the error for a simple lesion-deficit relation cannot be assumed to have an identical effect with more complex relations, indeed any effect at all. For example, one could trivially invert the error field depicted in Fig. 1 to “correct” a model, but this would *only* work were the lesion deficit relation really that simple. The challenge is to devise a method that succeeds with models of complexity far closer to reality—including two or more interacting areas—and is explicitly shown to do this (Mah et al., 2014, Pustina et al., 2017). Where a new approach is advocated, it is insufficient to stop at the first hurdle, examining only simple, single area models, as two of the published studies have done (Sperber and Karnath, 2017, Zhang et al., 2014).

Third, the point of synthetic modelling is to illuminate the optimal approach in the real world, not to come up with a solution specific to the synthetic context. One cannot, for example, bend the basic rules of inference. Sperber and Karnath (2017) arbitrarily prune the voxels of each modelled lesion at a proportional cuttoff—those affected in at least 5% of all lesions in any one study. Before any modelling proper begins, each lesion is distorted by removing voxels from it, to a degree and in a pattern that unpredictably interacts with their comparative rarity. Because the criterion is proportional, if one assembled a dataset of (say) 10,000 lesions one would *still* needlessly distort lesions in the same way, even though such voxels would now be sampled 500 times. To bring rare voxels back into the picture one would have to remove lesions confined to common areas, further distorting the lesion patterns fed into the model. Rarely hit voxels are, of course, generally further away from the vascular tree (where, as here, the aetiology is ischaemic), and so pruning them trivially reduces the mean localisation error. But to accept this is to assume that the function of areas of the brain varies with their vascular position, indeed that they might simply be *ignored* on that account alone. This is justifiable neither statistically not neuroanatomically, whatever any synthetic model might say. Any proposed solution must be inferentially viable in its real world application to be worth investigating at all.

**Fig. 2:**
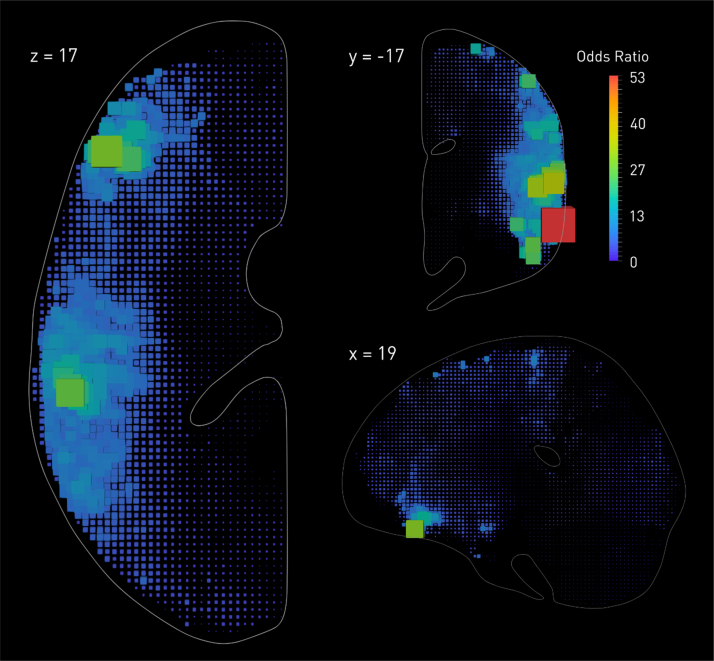
**The relation between lesion probability and overall lesion volume.** Reanalysing the data presented in Mah et al. (2014), here we used Bayesian logistic regression (Makalic and Schmidt, 2016), performed independently at each voxel, to estimate the odds ratio for the relation between the volume of a lesion and the probability of damage at each location across the brain. The odds ratios are visualised as box glyphs whose colour and dimensions are proportional to the estimated value. Note the enormous variation in the odds across the brain, and the complex anatomical pattern—distinct from the pattern observed in Fig. 1—it follows. Using lesion volume as a regressor in a mass-univariate lesion-deficit model will thus unquestionably distort the inference, penalising voxels more commonly hit by large lesions such as those that reach the cortical periphery or fall on vascular territorial boundaries. (For interpretation of the references to color in this figure legend, the reader is referred to the web version of this article.)

Fourth, it is helpful to remember that no methodological innovation can break the fundamental laws of physics. Where a distorting effect is generated by the complex interaction of *irreducibly many* variables—here the complex spatial features of lesions—it cannot be satisfactorily corrected by any *single* variable. For example, we know lesion volume could not explain the biasing effects we observe, for one could not thereby generate the error map shown in Fig. 1 or its inversion: the necessary information simply is not there. Of course, a single variable may well *modify* the pattern of distortion. Since lesion volume shows its own idiosyncratic variations with anatomy (see Fig. 2), it is *bound* to bias any low-dimensional spatial inference that incorporates it as a covariate. Areas of the brain showing strong correlations with lesion volume—generally those at the edges of vascular boundaries—will be unjustifiably penalised, whereas others just as unjustifiably favoured. For most voxels the error will be increased, for some it might be decreased because lesion volume gives the model some, even if incredibly crude, idea of collateral damage, but the effect overall will be to substitute one kind of distortion for a subtly differing other.

This is indeed what Inoue et al. (2014) found in their comprehensive simulation analysis. Sperber and Karnath (2017), by contrast, report a reduction in mislocalisation, and suggest regressing out lesion volume as a way of “correcting” mass univariate analyses. The *a priori* concerns aside, their approach is mathematically invalid, for they apply linear rather than logistic regression to a binary classification, rendering the residuals uninterpretable. Confidence in their calculations is further undermined by some of the vectors in their estimated error fields pointing outside the slice, where the estimated centroids could not physically lie (see Fig. 5 in their paper). In any event, where the proposed manoeuvre is information-theoretically powerless to correct the error, the value of any modelling is moot.

Fifth, although synthetic models have so far been used to quantify the fidelity of mapping behaviour onto anatomy, they can—and should—be used to quantify the fidelity of the inverse inference: from lesioned anatomy to behaviour (Godefroy et al., 1998). It is useful to be able to predict the behaviour of a patient—now, in the future, or in response to treatment—from the pattern of damage in conjunction with other clinical parameters. Since the behavioural effects are inevitably generated *through* the anatomy, one cannot ignore it, but it is here *implicit* in the model. Crucially, whether or not it is distorted need not affect the fidelity of any behavioural prediction, for all we need care about now is differentiating between lesions, not between the anatomical areas they disrupt.

To illustrate the point, consider a pathological process that *always* damages a neural locus that is itself of no consequence whatsoever. Damage to this locus will now be a “biomarker” with a sensitivity and specificity dependent wholly on the strength of the lesion-deficit association, not on any aspect of its specific function. Naturally, for the pathology to have a behavioural consequence it must also damage other, functionally critical, areas. But though our biomarker *cannot* be better than an index that correctly identifies all critical areas—logically cannot—it may offer a parsimonious description of their integrity that makes it reasonably and efficiently predictive. A simple analogy is the use of troponin in detecting a myocardial infarction: it could never be better than acute histology, but it is much more convenient to perform a blood test.

But none of this means lesion dimensionality may be ignored with impunity in this context, as some have argued (Price et al., 2017). In the next section, we briefly outline how the issue may be explored with synthetic models of lesion-deficit clinical prediction rather than lesion-deficit anatomical mapping.

## 6 Lesion-deficit models for clinical prediction

Consider two groups of lesioned patients differing in some behavioural parameter of interest such as response to rehabilitation. If the behavioural outcome is sensitive to the neuroanatomy, the lesion patterns in the two groups may differ. If so, a mass-univariate test will identify a set of discriminating voxels, which may or may not coincide with the causal neuroanatomy as the lesion bias dictates. To infer the behavioural outcome in an individual patient whose membership of the groups is unknown, we need to compare his lesion with the set of discriminating voxels, usually by calculating a (weighted) overlap between the two. The sensitivity and specificity of the classification then depends solely on how well the cluster of discriminating voxels reflects membership of the two groups. Parasitic voxels that are perfectly correlated with damage to critical voxels will naturally be no less predictive. But where strong lesion bias has displaced the estimated cluster far from the critical locus, thereby founding most of the prediction on parasitic voxels, lesions that happen to hit the critical locus *without* much collateral damage will now be misclassified. The relationship between lesion bias and classification performance thus becomes U shaped: best with zero or maximal lesion bias, worst somewhere inbetween.

**Fig. 3:**
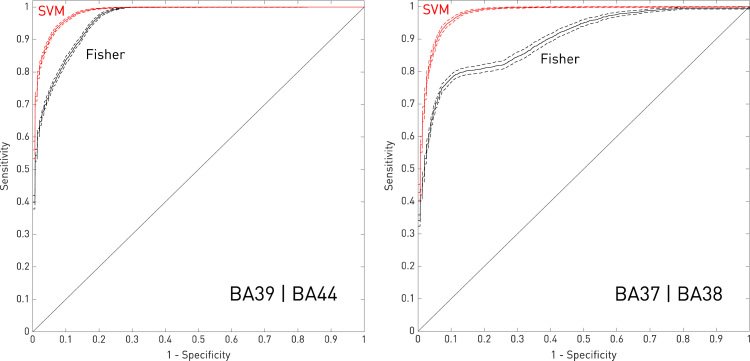
**Modelling clinical outcome prediction with low and high-dimensional methods.** Mean ROC curves(solid lines) and 95% confidence intervals (dotted lines) for predicting the disease label assuming a model of anatomical dependence involving damage to either BA39 or BA44 (left panel), or BA37 or BA38 (right panel), derived from the data reported in Mah et al. (2014). In the mass-univariate case (in black), the predictions were generated by applying a voxel-wise Fisher exact test to randomly sampled subsets of 407 lesions, thresholded at p < 0.001 uncorrected, and then testing on the remaining 174, generating a single predictive value for each test lesion using Fisher's method. In the high-dimensional multivariate case (in red), the predictions were generated by estimating the weighting of each voxel with a linear support vector machine (cost parameter= − 14), and applying the weights to each test image. The mean curves and their confidence intervals are derived from 200 random iterations across the full 581 lesion dataset. Note that the multivariate approach is markedly superior. (For interpretation of the references to color in this figure legend, the reader is referred to the web version of this article.)

To illustrate this, here we extended the two-region analyses described in Mah et al. (2014) to model the individual outcome predicted from the set of inferred critical voxels. As in the original paper, we posited a hypothetical syndrome probabilistically dependent on damage to at least 20% of the volume of either BA39 or BA44 and, in separate models, of either BA37 or BA38, with the probability set at 0.9 for those meeting the damage criterion. Taking randomly selected sets of 407 lesions (70% of the entire dataset), we proceeded to estimate the critical voxels with either a standard mass-univariate approach—voxelwise Fisher exact test—or a high-dimensional multivariate approach—linear support vector machines. We used the remaining 174 lesions to test the predictive performance of these maps, generating a separate ROC curve for each method from sets of possible thresholds of the estimated predictive parameters. To generate outcome predictions in the mass-univariate case, we used Fisher's method to derive a single negative log *p* value for each test case after voxel-wise multiplication of the lesion map to the Fisher exact test-estimated field of *p* values, generally thresholded at *p*<0.001 uncorrected so as not to remove the potentially helpful influence of voxels of low significance. In the multivariate case, we analogously applied the support vector machine-derived weights to each test lesion map. Iterating over 200 different randomisations of the data, we generated an average ROC curve for each inferential method, including 95% confidence intervals (Fig. 3).

It is easy to see the mass-univariate approach falls someway short of the performance achieved with a high-dimensional model. The AUC values for the support vector machine models are 0.979 (95%CI 0.977–0.981) for BA39|BA44, and 0.984 (95%CI 0.983–0.985) for BA37|BA38, whereas for the Fisher exact test models they are only 0.960 (95%CI 0.958–0.962) for BA39|BA44, and 0.900 (95%CI 0.893–0.907) for BA37|BA38. Note it is not the absolute numbers that matter here—these are idealised conditions—but the *relative difference* in performance, which clearly shows clinical outcome prediction needs anatomical fidelity too.

## 7 Implications

Lesion-deficit mapping has an enormously important role in systems neuroscience, not least in reigning in the rampant speculation fed by the perpetually “bull” market of functional imaging. No other method of comprehensive applicability across the brain has comparable inferential power, establishing the *necessity*—not just correlated activity—of a candidate neural substrate (Adolphs, 2016). But if it is to perform this role with authority its methodological foundations must be secure. The unavoidable practical difficulties of lesion mapping will generally place it at a numerical disadvantage against the weaker, correlative methods that are so much easier to deploy.

Here it is worth considering how evidential conflicts are naturally resolved in the field. In a perfect world, data would be stratified by quality—not quantity—and inferences from it commensurately weighted. But in the real world, the volume of published studies exerts a disproportionate influence on community beliefs. So where the behaviour can be positively defined or experimentally simulated—i.e. unlike visuospatial neglect—functional imagers will get to a problem faster than lesion mappers, in greater numbers, and will tend to dominate the discourse. Replicating a functional imaging finding with lesion mapping merely adds us to the also-rans, of distinctive interest only to our peculiar methodological clique. And obtaining a lesion mapping result that runs *counter* to the prevailing, functional imaging-driven models, no matter how compelling, is in practice far harder to sell than the data themselves might suggest. We wish to test the inferential foundations of lesion mapping so robustly here not out of a childish oppositionism to the past, but because our discipline needs the highest possible standards to overcome its numerical disadvantage.

Anyone in doubt about this should consider the case of the conflict detection model of the anterior cingulate (Botvinick et al., 2001), a highly influential theory of the role of this medial frontal region in the control of action, built on the now familiar conjunction of simple conceptual models with highly reductive behavioural paradigms substantiated with functional imaging data. Though torpedoed by nearly all lesion studies, holed underwater by conceptual considerations alone, the ship sails on, carried forward on the wind of correlative data. (The interested reader should see (Brown, 2011, Grinband et al., 2011, Nachev, 2011, Yeung et al., 2011) for a recent discussion). It helps, of course, that the anterior cingulate is rarely damaged (Mah et al., 2014), and its deep location makes it relatively inaccessible to other disruptive techniques such as TMS, but had the functional imaging predictions been rigorously lesion-tested at scale, perhaps less would have been unsafely invested in so obviously doomed a vessel.

The fieldcraft of cognitive neuroscience aside, the clinical applicability of lesion-deficit models is critically dependent on their individuating power. A clinician treats the specific patient before him or her: the group matters only as far as it informs that individual decision. Where individuality is richly multidimensional—as is certainly true of the brain—we cannot degrade one without degrading the other. But even those of us with less practical an interest ought to care, for any claim to mechanistic generality—the preoccupation of the scientist—will always be undermined by an alternative model with greater individuating power. It is no defence that the asserted model might be elegantly simple, for to the extent to which it does not fit predictable instances it cannot be adequately causal.

That the application of high-dimensional methods escalates the scale of the necessary delivery infrastructure is an opportunity at least as much as it is an obstacle. The investment will follow if we *justify* it: the kind of synthetic lesion-deficit modelling we have discussed can be used positively to show that high-dimensional methods can succeed as well as that low-dimensional methods fail. Rather than resisting criticisms of our current inferential framework we should use them to motivate its replacement with another, strong enough to deal with the challenges that lie ahead.

## Funding

This work was supported by the Wellcome Trust (WT-103709); the Department of Health (HICF-R9-501); and the UCLH NIHR Biomedical Research Centre.
